# *Labilibaculum manganireducens* gen. nov., sp. nov. and *Labilibaculum filiforme* sp. nov., Novel *Bacteroidetes* Isolated from Subsurface Sediments of the Baltic Sea

**DOI:** 10.3389/fmicb.2017.02614

**Published:** 2018-01-05

**Authors:** Verona Vandieken, Ian P. G. Marshall, Helge Niemann, Bert Engelen, Heribert Cypionka

**Affiliations:** ^1^Paleomicrobiology Group, Institute for Chemistry and Biology of the Marine Environment, University of Oldenburg, Oldenburg, Germany; ^2^Department of Bioscience, Center for Geomicrobiology, Aarhus University, Aarhus, Denmark; ^3^Aquatic and Stable Isotope Biogeochemistry, University of Basel, Basel, Switzerland; ^4^CAGE - Centre for Arctic Gas Hydrate, Environment and Climate, University of Tromsø, Tromsø, Norway; ^5^Departments of Marine Microbiology and Biogeochemistry, NIOZ Royal Netherlands Institute for Sea Research, ’t Horntje, and Utrecht University, Netherlands

**Keywords:** International Ocean Discovery Program, deep biosphere, Baltic Sea, salinity tolerance, iron and manganese reduction, fermentation

## Abstract

Microbial communities in deep subsurface sediments are challenged by the decrease in amount and quality of organic substrates with depth. In sediments of the Baltic Sea, they might additionally have to cope with an increase in salinity from ions that have diffused downward from the overlying water during the last 9000 years. Here, we report the isolation and characterization of four novel bacteria of the *Bacteroidetes* from depths of 14–52 m below seafloor (mbsf) of Baltic Sea sediments sampled during International Ocean Discovery Program (IODP) Expedition 347. Based on physiological, chemotaxonomic and genotypic characterization, we propose that the four strains represent two new species within a new genus in the family *Marinifilaceae*, with the proposed names *Labilibaculum manganireducens* gen. nov., sp. nov. (type strain 59.10-2M^T^) and *Labilibaculum filiforme* sp. nov. (type strains 59.16B^T^) with additional strains of this species (59.10-1M and 60.6M). The draft genomes of the two type strains had sizes of 5.2 and 5.3 Mb and reflected the major physiological capabilities. The strains showed gliding motility, were psychrotolerant, neutrophilic and halotolerant. Growth by fermentation of mono- and disaccharides as well as pyruvate, lactate and glycerol was observed. During glucose fermentation, small amounts of electron equivalents were transferred to Fe(III) by all strains, while one of the strains also reduced Mn(IV). Thereby, the four strains broaden the phylogenetic range of prokaryotes known to reduce metals to the group of *Bacteroidetes*. Halotolerance and metal reduction might both be beneficial for survival in deep subsurface sediments of the Baltic Sea.

## Introduction

*Bacteroidetes* are globally distributed and abundant in diverse environments from very energy-rich animals' guts to the most energy-limited habitats of our planet such as deep subsurface sediments, the deep biosphere (e.g., Biddle et al., 2008; Ley et al., 2008; Orsi et al., 2013; Inagaki et al., 2015). In the latter, microbes are cut off from the carbon supply of the water column as they are slowly buried in the sediment. Therefore, they conserve energy from the organic material in the sediment — organic material that becomes increasingly recalcitrant with depth (Jørgensen and Marshall, 2016). In addition to carbon limitation, changes in other environmental parameters with depth, such as temperature, pressure, redox state and the availability of terminal electron acceptors, are important constraints structuring microbial communities in the deep biosphere.

As oxygen is depleted within the uppermost sediment layers, fermentation becomes important for the breakdown of the recalcitrant organic material. Initially, more complex organic matter is broken down through fermentation to low molecular-weight molecules, which are further degraded in the terminal electron-accepting processes such as nitrate, manganese, iron and sulfate reduction and methanogenesis (Jørgensen, 1996). It has been shown that some fermenters can transfer a small part of their electron equivalents to iron oxides (Lovley, 2013). Thereby, they change the fermentative balance to a thermodynamically more favorable reaction (Lehours et al., 2010). These “fermentative iron reducers” typically transfer <5% of the electron equivalents from the substrates metabolized to iron (Lovley, 2013). However, it can be speculated that this still provides a competitive advantage for the fermenters of the deep biosphere which have to live on the scarce carbon sources present.

Besides the changes mentioned above, subsurface sediments of the Baltic Sea have experienced an increase in salt concentration by salinity changes of the overlying water column that started at the end of the last glaciation (Andrén et al., 2015). Consequently, seawater ions diffuse into deeper sediment layers, slowly increasing salinity during the last 9000 years. Thus, microbial communities in the sediment that originate from the time of rather low Baltic Sea salinity values are now exposed to increasing ion concentrations. As high ion concentrations exert physiological stress to prokaryotes, it has recently been shown by metagenome analyses, that the microbial communities present in deep sediment layers of the Baltic Sea have been structured by their ability to tolerate higher salinities (Marshall et al., in press).

Within the framework of the IODP Expedition 347, “Baltic Sea Paleoenvironment” our aim was to study salinity tolerance and adaptation to low-energy environments of Baltic Sea deep biosphere communities by cultivation-based studies. Here, we report on four new strains of the phylum *Bacteroidetes*, which were isolated from two coring sites from sediment depths of 14–52 mbsf with present-day salinities of 11–32. The strains represent two new species within a new genus. We present their physiological characterization and additionally analyzed the genomes of two strains concerning their energy metabolism and halotolerance in order to identify capabilities beneficial for life in deep subsurface sediments of the Baltic Sea.

## Material and methods

### Sampling sites, enrichment and isolation

Strains 59.10-1M, 59.10-2M and 59.16B were isolated at 15°C from sediment of IODP Expedition 347 to the Baltic Sea station M0059E (55°0.29′N, 10°6.49′E, Little Belt) and strain 60.6M from site M0060B (56°37.21′N, 11°40.24′E, Anholt Loch; Table 1). The sediment of site M0059E was drilled on 30 October 2013 and subsequently onboard sub-sampled into sterile plastic syringes, capped at the open side with a rubber stopper and transferred into gas-tight bags that were flushed with N_2_ before sealing. Samples were stored at 4°C until transport to the laboratory in Oldenburg, Germany, on 2 November 2013, where anoxic liquid enrichment cultures were immediately inoculated. The sediment of site M0060B was drilled on 29 September 2013, treated as described for M0059E and inoculated into media on 4 October 2013.

**Table 1:** Major physiological characteristics of new *Labilibaculum* strains, *M. albidiflavum* (Xu et al., 2016), *M. fragile* (Na et al., 2009), *M. flexuosum* (Ruvira et al., 2013) and *A. subtilis* (Wu et al., 2016).

	***L. filiforme* 59.10-1M**	***L. filiforme* 59.16B**	***L. filiforme* 60.6M**	***L. manganireducens* 59.10-2M**	***M. albidiflavum***	***M. fragile***	***M. flexuosum***	***A. subtilis***
Origin: site sediment depth (mbsf) salinity	Little Belt 32.2 20	Little Belt 51.5 11	Anholt L. 14.4 32	Little Belt 32.2 20	Coastal marine sediment	Tidal flat sediment Korea	Surface sea water Spain	Coastal marine sediment
Size of single cells width/length (μm)	0.6–0.7/3	0.6–0.8/2.7	0.5–0.7/2.2	0.6–0.8/0.9-3.8	0.3–0.4/2.5–15.0	0.5	0.3–0.4/2.6–30	0.3–0.4/2.9–30
Filament formation	+	+	+	−	+	+	+	+
Temperature range (optimum) (°C)	10–25 (20)	4–25 (25)	4–25 (25)	4–30 (30)	15–37 (33)	20–37.4 (33)	20–37 (20–30)	8–33 (28–30)
pH growth range (optimum)	5.9–8.7 (7.3–7.5)	6.4–8.7 (7.3–8.1)	6.5–8.0 (7.3–7.5)	5.9–8.0 (7.5–8.0)	6.5–9.0 (7.0–7.5)	6–8 (7)	ND	6.0–8.5 (7.5)
NaCl growth range (optimum) (%)	0–6.5 (1)	0–6.5 (0.5)	0.05–6.5 (1)	0.5–6.5 (1–2.5)	0.5–7.0 (2.0–3.0)	1–7 (3)	2–5	0.5–5.0 (2.0)
Fructose	−	−	−	+	ND	−	−	ND
Lactose	+	+	+	+	−	+	+	ND
Arabinose	−	−	−	+	ND	−	ND	ND
Xylose	+	+	+	+	ND	−	−	ND
Rhamnose	−	−	−	+	ND	ND	ND	ND
Cellobiose	+	+	+	+	+	−	+	−
Acetylglucosamine	+	+	+	+	ND	+	ND	+
Arginine	+	−	+	−	ND	ND	ND	ND
Glycerol	+	+	+	+	+	+	−	ND
Mn(IV) oxide reduction	−	−	−	+	ND	ND	ND	ND
G + C content (mol%) (based on genome sequencing)	35.6	35.8 (35.4)	36.4	36.7 (36.3)	43.8	45	32.9	36.5

*+, positive (for L. filiforme and L. manganireducens positive results were inferred from growth determined by optical density measurements in two consecutive transfers, for M. albidiflavum, M. fragile, M. flexuosum and A. subtilis positive results were inferred from acid production of API or Biolog tests), −, negative, ND, not determined. Glucose was used by all strains including M. fragile, M. flexuosum, M. albidiflavum and A. subtilis. Maltose was used by all strains including M. fragile but not determined for M. albidiflavum, M. flexuosum and A. subtilis. Galactose was used by all strains including M. fragile, M. albidiflavum and A. subtilis, not determined for M. flexuosum. Strains 59.10-1M, 59.10-2M, 59.16B and 60.6M additionally grew with pyruvate and lactate but did not use ribose, glutamine, asparagine, alanine, lysine, isoleucine, serine, histidine, betaine, succinate, fumarate, propionate, butyrate, malate, ethanol, butanol, butandiol, methanol, propanol, propandiol, mannitol, H_2_/CO_2_ (90/10, v/v), H_2_/CO_2_ + 0.5 mM acetate, peptone (0.1% w/v), tryptone (0.1% w/v), yeast extract (0.1% w/v) and casein (0.1% w/v) in the absence of an electron acceptor. The four strains reduced both Fe(III) citrate and Fe(III) oxides to Fe(II) but not nitrate to nitrite, nitrite or sulfate to sulfide in the presence of glucose. The four strains did not require vitamins for growth*.

The enrichments were started for strains 59.10-1M, 59.10-2M and 60.6M with artificial seawater medium and for strain 59.16B with brackish water medium both anoxic, pH buffered by a carbonate buffer and sulfide added as reducing agent as described previously (Vandieken et al., 2017). As carbon substrate, a monomer mix containing 36 different carbon sources (final concentration 0.1 mM each) was added (Vandieken et al., 2017). The strains were isolated in anoxic deep-agar dilution series. All further cultivation and tests were performed with the medium used for enrichment but with a single carbon source (for most analyses glucose in final concentration of 10–20 mM) instead of the monomer mix at 15°C.

### Characterization of the isolates

Transmission electron microscopy was performed with an EM 902A (Zeiss). For ultrathin sectioning, cells were fixed for 1 h in glutaraldehyde (3%) and formaldehyde (5%) and concentrated by centrifugation. The cell pellets were washed twice with phosphate-buffered saline (PBS) and subsequently stained with osmium solution for 1 h while shaking. Cells were again washed three times with PBS and afterwards dehydrated with increasing concentrations of ethanol (30–96%). The cells were incubated with different ratios of ethanol and Low Viscosity Embedding Media Spurr (Spurr, 1969) for 1–2 h (ethanol:Spurr mixture: 2:1, 1:1, 1:2, Spurr only). The Spurr was replaced by fresh Spurr before each incubation step: the samples were incubated overnight, then incubated at 40°C for 2 h and finally incubated at 70°C overnight. Ultrathin sections were cut with an Ultracut E microtome (Reichert, Germany). Physiological tests were performed in anoxic, liquid culture by glucose fermentation (if not indicated otherwise) at 15°C, and growth was determined by optical density measurements. Cultures grown with alternative substrates (final concentration 5–10 mM) were once again transferred to fresh medium to verify growth. Alternative electron acceptors were tested in the presence of glucose (10–20 mM). Nitrite concentrations (Grasshoff et al., 1999) and sulfide production (Cord-Ruwisch, 1985) were measured as described previously. Synthetic iron oxides were formed by neutralizing a 0.2 M FeCl_3_ solution to pH 7 with NaOH and subsequent dialysis of the iron oxide suspension. Fe^2+^ production from iron oxides and ferric citrate was tested in medium with and without sulfide by the ferrozine method (Stookey, 1970). Poorly crystalline manganese oxides were synthesized according to Lovley and Phillips (1988). Manganese oxide reduction was examined by the disappearance of brown manganese oxides in liquid culture. Mn^2+^ production was measured by flame atomic adsorption spectrometry. For tests on microaerophilic growth, cells were grown with 15 mM glucose in anoxic softagar and air in the headspace, so that the upper part of the agar shake was oxic (indicated by pink coloration) and the bottom part anoxic. Gram staining was performed by the standard Gram-staining technique. Catalase activity was inferred by the effect of 5% H_2_O_2_ (v/v) on a cell suspension, and oxidase activity was assessed according to the method of Kovacs (1956). NaCl requirement for growth was tested with 17 different NaCl concentrations between 0 and 7% (w/v). The pH ranges of the strains were determined in media with 12 different pH-values according to Knoblauch et al. (1999). The temperature ranges for growth were determined between 4 and 45°C. Growth tests for NaCl concentration, pH and temperature ranges were conducted with glucose (10 mM) as substrate. Products of fermentation were detected by high performance liquid chromatography with a refractive index detector (Graue et al., 2012). Fatty acids were analyzed by gas chromatography and quadrupole mass spectrometry according to previously published specifications (Blees et al., 2014). The G+C contents of genomic DNA were determined by HPLC at the DSMZ Germany.

### Sequencing and phylogenetic analysis of 16S rRNA genes

The 16S rRNA genes were amplified and sequenced with primers 8F, 518R and 1492R (Lane, 1991). Phylogenetic analysis was performed by the ARB program version arb-6.0.2 (Ludwig et al., 2004). The phylogenetic tree was calculated using maximum likelihood algorithm (PHYML with nucleotide substitution model HKY) and a 50% sequence conservation filter for *Bacteria*. Additionally, a maximum likelihood bootstrapping analyses was performed using RAxML rapid bootstrapping with 1,000 re-samplings.

### Genome sequencing and assembly for strains 59.10-2M and 59.16B

Genome sequencing was performed using the Nextera XT library preparation kit (Illumina, USA) and the Illumina MiSeq platform with a paired-end 300-bp MiSeq reagent kit version 3. The resulting sequencing reads were inspected for quality using FastQC version 0.10. (Andrews et al., 2011-2016) and trimmed for quality using Trimmomatic version 0.32 (Bolger et al., 2014): the first 20 bp and last 55 bp were removed, a quality-based trim was carried out with a minimum quality of 15 and a sliding window size of 4 bp, a minimum length of 200 bp was applied, and adapters were removed with parameters 2:30:10. Reads were assembled using spades version.3.6.1 (Bankevich et al., 2012) with automatic coverage cutoff, k-mers of 21, 33, 55, 77, 99, 127 and the “careful” parameter turned on. Coverage of each assembled scaffold was determined using bbmap version 35.x (Bushnell, 2010-2016), and scaffolds with <10-fold (strain 59.16B) and 15-fold (strain 59.10-2M) coverage were removed from the genomes. Genome completeness and contamination was assessed using CheckM version 0.9.4 (Parks et al., 2015). Genomes were submitted to the Integrated Microbial Genomes (IMG) platform and are accessible under taxon IDs 2648501602 (strain 59.16B) and 2648501603 (strain 59.10-2M). Assembled scaffolds were annotated using the IMG annotation pipeline (Huntemann et al., 2015). Sequencing of the genomic DNA for two strains yielded ~1.2 Gbp (0.7 Gbp post-trimming) for strain 59.10-2M and 1.5 Gbp (0.9 Gbp post-trimming) for strain 59.16B.

For the phylogenetic analysis using a concatenated protein alignment, protein sequences for each non-*Labilibaculum* genome in the alignment were obtained from GenBank in January 2017. In cases where multiple genome assemblies were available for a given species, the designated “representative assembly” was used. Concatenated ortholog alignments were created using quickCOAT (https://github.com/ianpgm/quickCOAT), which applied the following algorithm: blastp was used to search each genome's protein sequences against the genome for strain 59.10-2M. Hits with a bitscore 30% or more of the maximum possible bitscore (a cutoff used based on Lerat et al., 2003) exactly once in each genome were used to define single-copy orthologs. Each ortholog set was aligned using muscle with default settings (Edgar, 2004). Alignments were concatenated together and then trimmed using Gblocks (Talavera and Castresana, 2007). The trimmed alignment was 100X bootstrapped using Phylip SEQBOOT version 3.696, with phylogenetic trees constructed using FastTree version 2.1.9 (Price et al., 2010) according to the instructions for making bootstrapped trees in the FastTree documentation.

### Accession numbers

The GenBank accession numbers for the 16S rRNA gene sequences are KY509307 (strain 59.10-1M), KY509308 (strain 59.16B), KY509309 (strain 60.6M) and KY509310 (strain 59.10-2M). The Whole Genome Shotgun projects have been deposited at DDBJ/ENA/GenBank under accession MVDE00000000 (strain 59.10-2M) and MVDD00000000 (strain 59.16B).

## Results and discussion

### Genotypic and chemotaxonomic characterization

**Figure 1:**
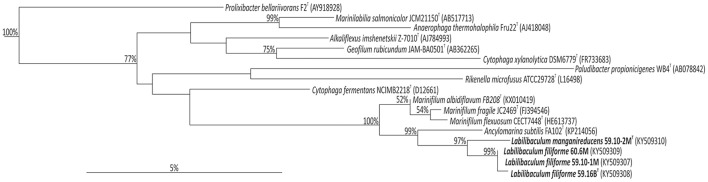
Phylogenetic tree based on 16S rRNA gene sequences of strains 59.10-1M, 59.10-2M, 59.16B and 60.6M and type strains of related species.

During a cultivation-based analysis of bacteria from Baltic Sea sediments sampled during IODP Expedition 347, we repeatedly isolated closely related *Bacteroidetes* strains. Strains 59.10-1M and 59.10-2M were isolated from the same sediment sample of Little Belt at 32 mbsf, while strain 59.16B was isolated from a deeper layer of the same site (52 mbsf) and a fourth strain, strain 60.6M, from the station at Anholt Loch and a depths of 14 mbsf (Table 1). The sequences of the 16S rRNA gene were identical for strains 60.6M and 59.10-1M and 99.3% similar to strain 59.16B, indicating that these three strains represent the same species. The fourth strain, strain 59.10-2M, had 97.4% identity to strain 59.16B and 97.0% to strains 60.6M and 59.10-1M based on 16S rRNA gene sequences. The closest cultivated relatives of the new strains were within the *Marinifilaceae* family comprising the genera *Marinifilum* and *Ancylomarina* (Table 2). The genus *Marinifilum* comprises three species, *M. fragile, M. flexuosum* and *M. albidiflavum*, which were isolated from tidal flat sediment, coastal surface water and costal marine sediment, respectively (Na et al., 2009; Ruvira et al., 2013; Xu et al., 2016), and the genus *Ancylomarina* has only the species *A. subtilis*, isolated from coastal marine sediment (Wu et al., 2016; Figure 1).

**Table 2:** Identity of 16S rRNA gene sequences of new *Labilibaculum* strains, 59.10-1M, 59.10-2M, 59.16B and 60.6M, *M. albidiflavum* (Xu et al., 2016), *M. fragile* (Na et al., 2009), *M. flexuosum* (Ruvira et al., 2013) and *A. subtilis* (Wu et al., 2016).

	***M. albidiflavum* (%)**	***M. fragile* (%)**	***M. flexuosum* (%)**	***A. subtilis* (%)**
*L. filiforme* 59.16B	94.0	93.1	93.3	94.2
*L. manganiredu-cens* 59.10-2M	94.4	93.3	93.9	93.9

**Figure 2:**
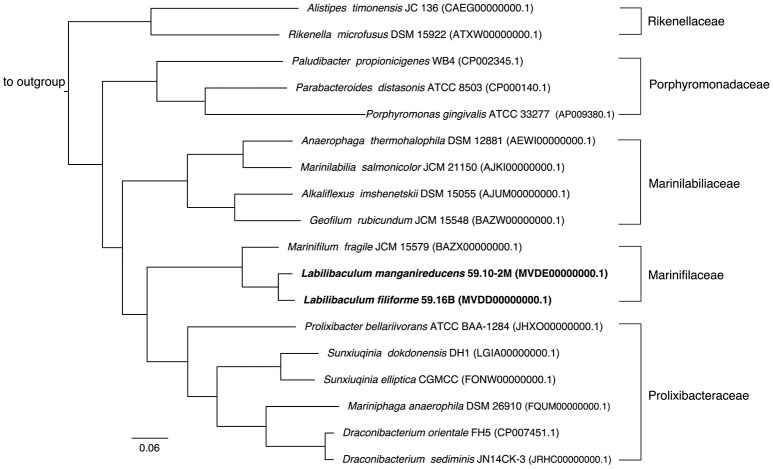
Phylogenetic tree constructed using a concatenated protein alignment from 213 single-copy orthologous genes with non-conserved regions trimmed, resulting in an alignment 56,521 amino acids wide. All species shown are from the class *Bacteroidia*, while the outgroup contains *Cytophaga aurantiaca* and *Cytophaga hutchinsonii* from the sibling class *Cytophagia*. All bootstrap values (not shown) are 100/100 (100%).

The draft genomes of strains 59.10-2M and 59.16B assembled into 121 and 62 contigs (>500 bp) with genome sizes of 5.3 and 5.2 Mb and an estimated completeness of 99.7 and 97%, respectively. The two genomes showed 82.5% average nucleotide identity for all genes, which is below the 94% identity considered for the differentiation at the species level (Konstantinidis and Tiedje, 2005). The concatenated protein tree based on the two genomes supported the phylogenetic placement of the new strains (Figure 2).

The dominant fatty acids (>9%) for the strains were i-C_15:0_, ai-C_15:0_ and C_15:0_, whereas *M. albidiflavum, M. fragile, M. flexuosum* and *A. subtilis* had more i-C_15:0_ fatty acids but fewer ai-C_15:0_ and C_15:0_ (Table 3). Interestingly, the analysis of the fatty acid composition also revealed methylated, unsaturated fatty acids, such as 12-methyl-C_15:1_Δ6, 12-methyl-C_15:1_Δ8 and 14-methyl-C_16:1_Δ8 to be abundant by 0.4–3.3%. These fatty acids have not been found in close relatives of the strains (Ruvira et al., 2013). Indeed they are rather unusual for bacteria in general. Methylated fatty acids such as 10-methyl-C_16:1_Δ7 have been detected in highly enriched cultures of the methane-oxidizing, nitrite-reducing bacterium *Candidatus* Methylomirabilis oxyfera, while 10-methyl-C_16:1_Δ8 was found in anaerobically methane-oxidizing microbial mats from the Black Sea (Blumenberg et al., 2004; Kool et al., 2012).

**Table 3:** Fatty acid abundance in relative proportions of new *Labilibaculum* strains, 59.10-1M, 59.10-2M, 59.16B and 60.6M, *M. albidiflavum* (Xu et al., 2016), *M. fragile* (Na et al., 2009), *M. flexuosum* (Ruvira et al., 2013) and *A. subtilis* (Wu et al., 2016).

	***L. filiforme* 59.10-1M**	***L. filiforme* 59.16B**	***L. filiforme* 60.6M**	***L. manganireducens* 59.10-2M**	***M. albidiflavum***	***M. fragile***	***M. flexuosum***	***A. subtilis***
i-C_14:0_						0.5		0.2
C_14:0_	0.7	0.6	0.3	0.6	0.4	0.2	3.4	0.3
i-C_15:1_ω8	5.0	12.4	11.5	9.8				
ai-C_15:1_ω8	1.7	4.4	3.2	3.1				
i-C_15:0_	17.8	23.2	29.3	20.7	50.7	56.2	41.3	43.8
ai-C_15:0_	17.5	14.5	17.3	12.1	0.6	3.5	1.8	5.2
C_15:1_ω8*c*	3.3	3.0	2.5	3.6				
C_15:1_ω6*c*	4.8	5.7	4.7	5.5				
C_15:1_ω4	0.3	0.4	0.3	0.4				
C_15:0_	12.6	11.0	9.4	13.0		0.1		0.1
12-methyl-C_15:1_Δ6	1.3	2.2	1.7	2.2				
12-methyl-C_15:1_Δ8	1.9	3.3	2.9	3.0				
ai-C_16:0_	1.4	1.6	1.2	1.7				
C_16:1_ω9*c*	0.8	0.9	0	0.6				
C_16:1_ω7*c*	0	0.1	0.4	0				
C_16:1_ω5*c*	0.1	0.1	0	0.1				
C_16:0_	4.3	1.4	0.8	3.4	0.5	0.3	5.6	0.3
i-C_17:1_ω9*c*					10.0			4.9
i-C_17:1_ω8	2.5	4.7	7.2	4.6				
ai-C_17:1_ω8	0.4	0.8	0.8	0.9				
14-methyl-C_16:1_Δ8	0.4	0.8	1.0	0.6				
i-C_17:0_	0.4	0.3	0.2	0.3	1.6			0.3
C_17:1_ω8	2.4	2.1	1.9	2.7				
C_17:1_ω6	3.3	2.5	2.1	3.1				
C_17:0_	0.5	0.3	0.1	0.4				
C_18:0_	1.0	0.2	0.2	0.4	0.2		3.5	0.1
C_18:1_ω9*c*	10.1	1.9	0.8	3.4	0.6		2.5	
C_18:1_ω8*c*	0.9	0.2	0.1	0.4				
C_18:1_ω7*c*	0.6	0.1	0	0.2				
C_18:1_ω5*c*					0.2		2.1	0.5
C_18:0_	4.0	1.1	0.3	3.5	0.2			0.1

### Phenotypic characterization and physiological range

**Figure 3:**
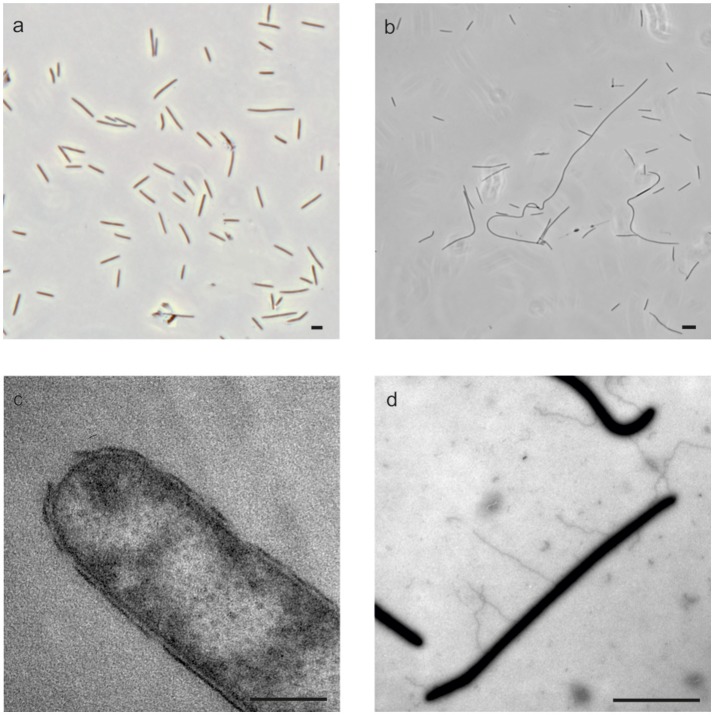
Morphology of the cells. Phase-contrast micrographs of strain 59.10-2M showing single cells **(a)** and strain 59.16B showing formation of filaments **(b)**. TEM micrographs of ultrathin sections of strain 59.10-1M showing Gram-negative cell wall **(c)** and of strain 59.16B showing appendages **(d)**. Scale bars 5 μm for **(a,b,d)** and 0.2 μm for **(c)**.

All strains grew as rods with sizes of 0.6 × 2.2–3.8 μm, and strains 59.10-1M, 59.16B and 60.6M also formed filaments of lengths up to 100 μm (Table 1, Figures 3a,b). The four strains stained Gram-negative, which was confirmed by electron micrographs of ultrathin sections showing the characteristic two membranes of Gram-negative bacteria (Figure 3c). All cells produced appendages with diameters of 30–40 nm (Figure 3d). Cells and filaments showed a gliding motility with occasional flips, which are characteristic for *Bacteroidetes* cells (McBride and Zhu, 2013). Consistent with the observation of gliding movement, genes for gliding motility were found in the genomes, while flagella genes were not detected (Supplmentary Table 1). Recently, RNA transcripts of genes for gliding motility were found in different depths of Baltic Sea sediment Station M0059 by metatranscriptome analysis (Zinke et al., 2017).

All strains were catalase and oxidase negative. The strains were psychrotolerant, growing at 4°C (except strain 59.10-1M) with optimum temperatures for growth of 20–25°C, only strain 59.10-2M had a higher optimum of 30°C (Table 1). All strains were neutrophilic and showed a broad tolerance toward NaCl concentrations (0.5–6.5%; Table 1). Optimum NaCl concentrations were 0.5 and 1%, only strain 59.10-2M showed a broader NaCl concentration optimum of 1–2.5%. In the genomes, the halotolerance of the strains was indicated by the presence of Na^+^/H^+^ antiporter, transporter systems for osmolytes (betaine, choline, glycine, or proline), chaperones and other genes that might enable the strains to grow at high salt concentrations (Supplementary Table 1) (Waditee et al., 2002; Wood, 2015). In a recent study of metagenomes from sediments sampled during IODP Expedition 347 to the Baltic Sea, the abundance of Na^+^/H^+^ antiporter genes correlated to the present salinity of the respective sediment sample (Marshall et al., in press). As sediments of the Baltic Sea have experienced an increase in salinity by diffusion of ions into deep sediments with low salinity during the last 9000 years, the data indicated that microbial community structures of deep sediments have been shaped by their ability to tolerate higher salinities. The physiological tests revealed a broad tolerance for salinity (limnic to marine conditions) for all four isolates. The different salinities in the sediments of the isolates' origin (11–32; Table 1) showed that the strains are able to cope with changes in salt concentrations and thus to grow in deep subsurface sediments of the Baltic Sea.

### Fermentative metabolism

All strains grew by fermentation of various mono- and disaccharides (e.g., glucose, galactose, xylose and maltose) as well as pyruvate, lactate and glycerol (Table 1). Strain 59.10-2M additionally fermented fructose, arabinose and rhamnose. The main end products of glucose fermentation were formate > acetate > succinate with minor amounts of propionate and malate for strains 59.10-1M, 59.16B and 60.6M, while strain 59.10-2M produced more propionate than succinate. Genes for the central metabolic pathways were found in both genomes including glycolysis, the TCA cycle as well as the reductive TCA cycle supporting the capability of the strains to ferment glucose (Supplementary Table 1). However, autotrophic growth was not found in physiological tests with H_2_/CO_2_ as electron donor and carbon source (with and without 0.5 mM acetate). Propionate was likely formed via the methylmalonyl-CoA pathway (Supplementary Table 1). The ability to use pyruvate as substrate was confirmed by the presence of a pyruvate-formate lyase, which yields acetate and formate, and a pyruvate-ferredoxin oxidoreductase which yields acetate, CO_2_ and H_2_ (Supplementary Table 1). Furthermore, fermentation of lactate, glycerol, acetylglucosamine, xylose, galactose, cellobiose, maltose and lactose was confirmed by the presence of genes encoding their respective degradation pathway (Supplementary Table 1). The additional physiological capabilities of strain 59.10-2M, such as the degradation of arabinose and rhamnose, could be supported by the presence of the respective genes only being present in the genome of strain 59.10-2M (Supplementary Table 1). On the other hand, the difference in the capacity to use fructose between the two strains was not identified in the genomes, as both strains possess genes that encode for phosphofructokinase and glycolysis (Supplementary Table 1). Overall, the degradation of mono- and disaccharides is in line with the frequent finding that members of the *Bacteroidetes* are involved in polysaccharide degradation in seawater and sediments (e.g., Fernández-Gómez et al., 2013; Sun et al., 2016).

### Usage of terminal inorganic electron acceptors

The physiological behavior toward oxygen differed between the strains. During growth in anoxic deep agar dilution culture with air in the headspace, strain 59.10-2M preferentially grew at the surface of the tube where oxygen was diffusing into the agar, whereas the other three strains only grew in the anoxic bottom of the tube, i.e., not in the upper oxic zone (Supplementary Figure A.1). These observations suggested that strain 59.10-2M tolerated oxygen and/or grew by aerobic respiration during microaerophilic conditions, whereas the other three strains only grew in deeper anoxic agar layers, most likely by fermentation. Genes for oxygen detoxification were identified in both genomes including the superoxide reductase (the system of anaerobic prokaryotes) as well as genes of aerobic prokaryotes which produce O_2_ during detoxification, the superoxide dismutase and catalase (Supplementary Table 1). Additionally, both strains encoded a *cbb3*-type cytochrome *c* oxidase comprised of three subunits as well as a second cytochrome *c* oxidase (Cox; Supplementary Table 1). Both enzymes function as terminal oxygen reductase and have been suggested to be associated with microaerophilic growth (Ramel et al., 2013; Mardanov et al., 2016). The third system for terminal oxygen reduction, the cytochrome *bd*-type quinol oxidase with its two subunits, however, was present only in strain 59.10-2M (Supplementary Table 1). This might explain the difference in microaerophilic growth of the two strains. For example, deletion mutations of this gene increased oxygen sensitivity in *Desulfovibrio vulgaris* (Ramel et al., 2013).

**Figure 4:**
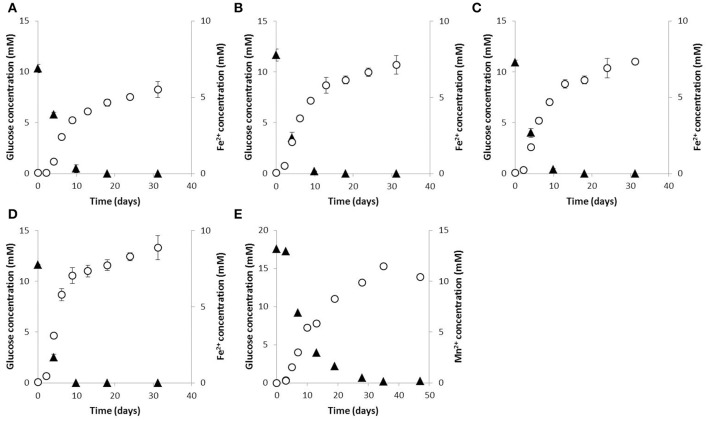
Iron and manganese reduction during glucose fermentation. Glucose consumption (triangles) and production of Fe^2+^ or Mn^2+^ (circles) of **(A)** strain 59.10-1M, **(B)** strain 59.16B, **(D)** strain 60.6M and **(D)** strain 59.10-2M grown with Fe(III) oxides and **(E)** strain 59.10-2M grown with Mn(IV) oxides.

Reduction of sulfate to sulfide, nitrate to nitrite or nitrite was not observed in the presence of glucose. Interestingly, dissolved Fe^3+^ as well as Fe(III) oxides were reduced during glucose fermentation, while strain 59.10-2M also slowly reduced Mn(IV) oxides over several weeks of cultivation (Figure 4). Manganese reduction by strain 59.10-2M was additionally observed with lactate as electron donor but not with ethanol and acetate. Strains 59.10-1M, 59.16B and 60.6M transferred 2–3% of electron equivalents from glucose to Fe(III) oxides (Figures 4A–C), and 6–12% of electrons to dissolved Fe(III) citrate. Strain 59.10-2M transferred 16, 4 and 6% of electron equivalents to Fe(III) citrate, Fe(III) oxides and Mn(IV) oxides, respectively, during glucose fermentation (Figures 4D,E). Thus, the strains represent the “fermentative metal reducer type,” which transfers only a small part of the electron equivalents to the metal (typically <5%) while most of the electron equivalents are recovered in fermentation products (Lovley, 2013). However, even if only a small part of electron equivalents was transferred to iron oxides, it has been shown to improve the fermentative balance (less formation of acetate, butyrate, or hydrogen) resulting in thermodynamically more favorable conditions (Lehours et al., 2010; Dong et al., 2016). Thus, the available substrates were used more efficiently by the fermenter, which might be an important competitive advantage in carbon-limited environments like the deep biosphere.

The capability of iron and manganese reduction has rarely been tested for *Bacteroidetes* species, and only one species, *Bacteroides hypermegas*, appears in the list of iron-reducing organisms (Jones et al., 1984; Lovley, 2013). Bacteria of the deep biosphere have been shown previously to reduce metals including members of the *Firmicutes, Proteobacteria, Deferribacteres* and *Deinococcus-Thermus* (Roden and Lovley, 1993; Greene et al., 1997; Kieft et al., 1999; Dong et al., 2016; Vandieken et al., 2017), supporting the suggestion that iron and manganese oxides in Baltic Sea sediments (Hardisty et al., 2016) might be reduced by fermenters in order to more efficiently use the scarce organic material for survival in the deep biosphere.

The terminal transfer of electrons to Fe(III) has been studied in detail only for organisms that transfer most of their electron equivalents during growth to Fe(III) as terminal electron acceptor. The best studied genera are the gammaproteobacterial *Shewanella* and the deltaproteobacterial *Geobacter* where iron reduction was found to require *c*-type cytochromes (Waditee et al., 2002; Weber et al., 2006). Besides the above mentioned cytochrome *c*-containing enzymes that might be used for the terminal reduction of oxygen, the genomes of strains 59.10-2M and 59.16B contain a predicted ferric reductase and cytochrome *c* annotated as nitrite reductase (NrfHA, Supplementary Table 1). This gene has also been identified in the genome of the metal-reducing *Desulfotomaculum reducens* which—just as our two strains—was not able to reduce nitrite (Junier et al., 2010). Hence, in line with Junier et al. (2010), we conclude that the annotation for nitrite reductase might not be correct but instead the cytochrome might be used for the reduction of U(VI) or Fe(III) by *D. reducens*. In order to specify the genes involved in manganese and iron reduction, more detailed studies are needed specifically for the “fermentative type” of metal reducers, as it has been shown that the electron transport pathways for the well-studied metal reducing genera of gammaproteobacterial *Shewanella* and deltaproteobacterial *Geobacter* already differ considerably (Melton et al., 2014).

### Proposal for a new genus *Labilibaculum* with two new species

From the genera *Marinifilum* and *Ancylomarina* species of the new genus *Labilibaculum* can be differentiated by their gliding motility ability, while *Marinifilum* and *Ancylomarina* species are non-motile (Wu et al., 2016; Xu et al., 2016). Furthermore, species of *Labilibaculum* were not able to grow at fully oxygenated conditions, whereas species of *Marinifilum* and *Ancylomarina* were facultative anaerobic (Wu et al., 2016). From the genus *Marinifilum*, the new strains of the new genus *Labilibaculum* differed by being psychrotolerant, whereas the genus *Ancylomarina*, which so far comprises only the species *A. subtilis*, is also psychrotolerant (Table 1). Although direct comparison with species of *Marinifilum* and *Ancylomarina* is limited due to the lack of physiological tests that were performed under identical conditions, our data already provide evidence that the new strains differ based on phenotype and physiology. Due to these differences and the 16S rRNA gene identity of <94.5% (Yarza et al., 2014) to species of both genera, *Marinifilum* and *Ancylomarina*, the establishment of a new genus is proposed. Strain 59.10-2M showed distinct physiological features to represent a second species within the new genus *Labilibaculum*. Compared to strains 59.10-1M, 59.16B and 60.6M, strain 59.10-2M exhibited manganese oxide reduction, fermentation of a broader selection of substrates, different products of glucose fermentation, broader salinity optimum, inability to grow at freshwater conditions, growth at higher temperatures and no formation of filaments (Table 1). Additionally, 16S rRNA gene sequence similarities of 97.0–97.4% of strain 59.10-2M to strains 59.10-1M, 59.16B and 60.6M, and the overall genome similarity of 82.5% average nucleotide identity to strain 59.16B indicated the distinction of the two strains on the species level. In conclusion the results support the proposal of two new species inside a new genus, as we propose *Labilibaculum manganireducens* gen. nov., sp. nov. (type strain 59.10-2M^T^) and *Labilibaculum filiforme* sp. nov. (type strains 59.16B^T^, additional strains of this species 59.10-1M and 60.6M).

### Description of *Labilibaculum* gen. nov.

*Labilibaculum* (La.bi.li.ba'cu.lum: L. adj. *labilis* flexible, gliding; L. neut. n., *baculum* a rod; L. neut. n. *Labilibaculum* a gliding rod).

Straight, long and thin rods. Gliding motility with occasional flips. Gram-stain-negative and some strains form filaments. Psychrotolerant. Heterotrophic fermentation. Major fatty acids are i-C_15:0_, ai-C_15:0_ and C_15:0_. The genomic DNA G + C content is 35.0–37.0 mol%. The type species is *L. manganireducens*.

### Description of *Labilibaculum manganireducens* sp. nov.

*Labilibaculum manganireducens* (man.ga.ni re.du'cens. N.L. n. *manganum* manganese; L. part. adj. *reducens* in chemistry, converting to reduced state; N.L. part. adj. *manganireducens* reducing manganese).

Cells are 0.6–0.8 × 0.9–3.8 μm. Psychrotolerant, growth range of 4–30°C, with optimal growth at 30°C. The growth range for pH is 5.9–8.0 (optimum pH 7.5–8.0) and for NaCl 0.5–6.5% (optimum 1–2.5%). Fermentation of fructose, glucose, arabinose, cellobiose, acetylglucosamine and rhamnose. Fe(III) and Mn(IV) oxides are reduced as electron acceptors in the presence of glucose. Microaerophilic growth. The DNA G + C content of the type strain is 36.7 mol%. The type strain 59.10-2M^T^ (= DSM 102944^T^ = JCM 31100^T^) was isolated from the subsurface of marine sediment from Little Belt (Baltic Sea).

### Description of *Labilibaculum filiforme* sp. nov.

*Labilibaculum filiforme* (fi.li.for'me. L. n. *filum* a thread; L. suff. *forme* of the shape of; N.L. neut. adj. *filiforme* thread-shaped).

Cells are 0.5–0.8 × 2.2–3 μm and formation of filaments. Psychrotolerant, growth range of 10° (for some strains 4°) to 25°C, with optimal growth at 20–25°C. The growth range for pH is 6.5–8.0 (optimum around pH 7.4) and for NaCl 0.05–6.5% (optimum 0.5–1%). Fermentation of glucose, cellobiose, acetylglucosamine and some strains arginine. Fe(III) oxides are reduced as electron acceptors in the presence of glucose but not Mn(IV) oxides. The DNA G + C content of the type strain is 35.8 mol%. The type strain 59.16B (= DSM 101180^T^ = JCM 31101^T^) was isolated from the subsurface of marine sediment from Little Belt (Baltic Sea). Other strains of this species are 59.10-1M (= DSM 101179 = JCM 31099) and 60.6M (= DSM 101181 = JCM 31102).

## Author contributions

VV: Designed the study and performed most of the laboratory work and data analysis; IM: Performed most of the genome analysis; HN: Performed the fatty acid analysis; VV: Wrote the manuscript; VV, IM, HN, BE and HC: Edited the manuscript.

### Conflict of interest statement

The authors declare that the research was conducted in the absence of any commercial or financial relationships that could be construed as a potential conflict of interest.

## Abbreviations

msbf: meters below seafloor
IODP: International Ocean Discovery Program
IMG: Integrated Microbial Genomes.
