# Supplementary material for: Labilibaculum manganireducens gen. nov., sp. nov. and Labilibaculum filiforme sp. nov., Novel Bacteroidetes Isolated from Subsurface Sediments of the Baltic Sea
Source: Front Microbiol. 2018 Jan 5;8:2614. doi: 10.3389/fmicb.2017.02614 (PMC5760507; doi:10.3389/fmicb.2017.02614)
Supplement: Supplementary file 1 [file DataSheet1.PDF]

Supplementary Material

Labilibaculum manganireducens gen. nov., sp. nov. and *Labilibaculum filiforme* sp. nov., novel *Bacteroidetes* isolated from subsurface sediments of the Baltic Sea

Verona Vandieken*, Ian P. G. Marshall, Helge Niemann, Bert Engelen, Heribert Cypionka

*** Correspondence:** Verona Vandieken: vvandieken@daad-alumni.de

1 Supplementary Figures and Tables

1.1 Supplementary Table

Supplementary Table 1. Annotation of genes for strain 59.16B (IMG genome ID 2648501602, DDBJ/ENA/GenBank ID MVDD00000000) and strain 59.10-2M (IMG genome ID 2648501603, DDBJ/ENA/GenBank ID MVDE00000000)

Name	ID	Presence in genome		IMG locus tags		NCBI locus tags	
		59.16B	59.10-2M	59.16B	59.10-2M	59.16B	59.10-2M
**Gliding**							
Gliding motility-associated ABC transporter ATP-binding subunit GldA	TIGR03522	+	+	Ga0104931_107195	Ga0104932_103136	BZG02_11770	BZG01_16530
Gliding motility-associated lipoprotein GldB	TIGR03514	+	+	Ga0104931_102448	Ga0104932_104215	BZG02_04845	BZG01_18815
Gliding motility-associated lipoprotein GldD	TIGR03512	+	+	Ga0104931_101271	Ga0104932_1002185	BZG02_01325	BZG01_02250
Gliding motility-associated protein GldE	TIGR03520	+	+	Ga0104931_101270	Ga0104932_1002186	BZG02_01320	BZG01_02255
Gliding motility-associated ABC transporter permease protein GldF	TIGR03518	+	+	Ga0104931_102138	Ga0104932_100972	no_NCBI_match	no_NCBI_match
Gliding-associated putative ABC transporter substrate-binding component GldG	TIGR03521	+	+	Ga0104931_101441<br>Ga0104931_102137	Ga0104932_100220<br>Ga0104932_100971	BZG02_02180<br>no_NCBI_match	BZG01_01430<br>no_NCBI_match
Gliding motility-associated lipoprotein GldH	TIGR03511	+	+	Ga0104931_107119	Ga0104932_102261	BZG02_11405	BZG01_14245
Gliding motility-associated lipoprotein GldJ	TIGR03524	+	+	Ga0104931_101155	Ga0104932_1005165	BZG02_00755	BZG01_05625
Gliding motility-associated lipoprotein GldK	TIGR03525	+	+	Ga0104931_107156	Ga0104932_102225	BZG02_11585	BZG01_14075
Gliding motility-associated protein GldL	TIGR03513	+	+	Ga0104931_107157	Ga0104932_102224	BZG02_11590	BZG01_14070
Gliding motility-associated protein GldM	TIGR03517	+	+	Ga0104931_107158	Ga0104932_102223	BZG02_11595	BZG01_14065
Gliding motility associated protien GldN	TIGR03523	+	+	Ga0104931_107159	Ga0104932_102222	BZG02_11600	BZG01_14060
Cell surface protein SprA	TIGR04189	+	+	Ga0104931_10729	Ga0104932_101525	BZG02_10955	BZG01_11085

**Osmoregulation**								
	Na+/H+ antiporter NhaD or related arsenite permease	COG1055	+	+	Ga0104931_10160<br>Ga0104931_11618	Ga0104932_101739	BZG02_00290<br>BZG02_16410	BZG01_12115
	Na+/H+ antiporter NhaC	COG1757	+	+	Ga0104931_101190<br>Ga0104931_101208	Ga0104932_1002260<br>Ga0104932_105023	BZG02_00930<br>BZG02_01010	BZG01_02620<br>BZG01_20045
	Na+/H+ antiporter NhaA	COG3004	+	+	Ga0104931_10423	Ga0104932_100160<br>Ga0104932_102520	BZG02_06955	BZG01_00300<br>BZG01_14945
	Small conductance mechanosensitive channel	KO:K03442	+	+	Ga0104931_104134<br>Ga0104931_10990	Ga0104932_102960	BZG02_07505<br>BZG02_13300	BZG01_16150
	Miniconductance mechanosensitive channel	KO:K16053	+	+	Ga0104931_102372	Ga0104932_102842	BZG02_04460	BZG01_15780
	Trehalose-phosphatase	TIGR00685	+	+	Ga0104931_106218	Ga0104932_102338	BZG02_10615	BZG01_14470
	MIP family channel proteins, glycerol uptake facilitator protein	TIGR00861	+	+	Ga0104931_10998	Ga0104932_103351	BZG02_13330	BZG01_17085
	Choline-glycine betaine transporter	COG1292	+	-	Ga0104931_11644	-	BZG02_16540	-
	Choline-glycine betaine transporter	COG2113	-	+	-	Ga0104932_100871	-	BZG01_07320
	ABC-type proline/glycine betaine transport system	COG4175	-	+	-	Ga0104932_100869	-	BZG01_07310
	ABC-type proline/glycine betaine transport system	COG4176	-	+	-	Ga0104932_100870	-	BZG01_07315
	Molecular chaperone DnaK (HSP70)	COG0443	+	+	Ga0104931_101111	Ga0104932_104123	BZG02_00550	BZG01_18640
**Glycolysis**								
	ADP-dependent phosphofructokinase/glucokinase	COG4809	+	+	Ga0104931_11912	Ga0104932_102068	BZG02_17410	BZG01_13555
	Glucose-6-phosphate isomerase	COG0166	+	+	Ga0104931_101468	Ga0104932_10278	BZG02_02310	BZG01_15345
	Fructose-6-phosphate kinase	COG0205	+	+	Ga0104931_102153<br>Ga0104931_103193<br>Ga0104931_11652	Ga0104932_100331<br>Ga0104932_100989<br>Ga0104932_102120	BZG02_03420<br>BZG02_05865<br>BZG02_16580	BZG01_02835<br>BZG01_08095<br>BZG01_13720
	Fructose-1,6-bisphosphatase	COG0158	+	+	Ga0104931_12138	Ga0104932_101022	BZG02_18145	BZG01_08335
	Fructose bisphosphate aldolase	COG0191	+	+	Ga0104931_12227	Ga0104932_100578	BZG02_18360	BZG01_05200
	Triose phosphate isomerase	COG0149	+	+	Ga0104931_103178	Ga0104932_100316	BZG02_05790	BZG01_02760
	Glyceraldehyde-3-phosphate dehydrogenase	COG0057	+	+	Ga0104931_12240	Ga0104932_100591	BZG02_18425	BZG01_05265

	Phosphoenolpyruvate synthase	COG0574	+	+	Ga0104931_102265	Ga0104932_1005135	BZG02_03940	BZG01_05475
	3-phosphoglycerate kinase	COG0126	+	+	Ga0104931_107122	Ga0104932_102258	BZG02_11420	BZG01_14230
	Phosphoglycerate mutase	COG0696	+	+	Ga0104931_12027	Ga0104932_101275	BZG02_17790	BZG01_09810
	Enolase	COG0148	+	+	Ga0104931_101288	Ga0104932_1002165	BZG02_01410	BZG01_02150
	Pyruvate kinase	COG0469	+	+	Ga0104931_1054<br>Ga0104931_10963	Ga0104932_102933	BZG02_08225<br>BZG02_13170	BZG01_16015
	Alcohol dehydrogenase	COG1454	-	+	-	Ga0104932_102644<br>Ga0104932_100778	-	BZG01_15270<br>BZG01_06725
	NADP-dependent alcohol dehydrogenase	COG1979	+	+	Ga0104931_11183	Ga0104932_104021	BZG02_14715	BZG01_18430
**Citric acid cycle**								
	Citrate synthase	COG0372	+	+	Ga0104931_103327	Ga0104932_1003178<br>Ga0104932_10262	BZG02_06520	BZG01_03580<br>BZG01_15070
	Aconitate hydratase	TIGR00117	+	+	Ga0104931_12241	Ga0104932_100592	BZG02_18430	BZG01_05270
	Isocitrate dehydrogenase	COG0473	+	+	Ga0104931_106149<br>Ga0104931_13119	Ga0104932_100673<br>Ga0104932_103236	BZG02_10270<br>BZG02_20145	BZG01_06055<br>BZG01_16805
	2-oxoglutarate dehydrogenase	COG0567	+	+	Ga0104931_102179	Ga0104932_1009114	BZG02_03540	BZG01_08225
	Succinyl-CoA synthetase, beta subunit	COG0045	+	+	Ga0104931_10757	Ga0104932_101553	BZG02_11095	BZG01_11230
	Succinyl-CoA synthetase, alpha subunit	COG0074	+	+	Ga0104931_10758	Ga0104932_101554	BZG02_11100	BZG01_11235
	Succinate dehydrogenase/fumarate reductase, Fe-S protein subunit	COG0479	+	+	Ga0104931_12417	Ga0104932_10136	BZG02_18870	BZG01_10010
	Succinate dehydrogenase/fumarate reductase, flavoprotein subunit	COG1053	+	+	Ga0104931_109116<br>Ga0104931_12415	Ga0104932_10138	BZG02_13410<br>BZG02_18860	BZG01_10020
	Tartrate dehydratase alpha subunit/Fumarate hydratase class I	COG1951	+	+	Ga0104931_104123	Ga0104932_100840	BZG02_07450	BZG01_07155
	Fumarate hydratase, class II	COG0114	-	+	-	Ga0104932_101628	-	BZG01_11590
	Isocitrate/isopropylmalate dehydrogenase	COG0473	+	+	Ga0104931_106149<br>Ga0104931_13119	Ga0104932_100673<br>Ga0104932_103236	BZG02_10270<br>BZG02_20145	BZG01_06055<br>BZG01_16805
	Malate dehydrogenase, NAD-dependent	TIGR01763	+	+	Ga0104931_11425	Ga0104932_101245	BZG02_15750	BZG01_09665

**Reverse citric acid cycle** (additional genes to citric acid cycle)								
	2-oxoglutarate/2-oxoacid ferredoxin oxidoreductase subunit alpha	KO:K00174	+	+	Ga0104931_101224<br>Ga0104931_102203	Ga0104931_102203<br>Ga0104932_100518	BZG02_01095<br>BZG02_03645	no_NCBI_match<br>BZG01_04900
	2-oxoglutarate/2-oxoacid ferredoxin oxidoreductase subunit beta	KO:K00175	+	+	Ga0104931_101223<br>Ga0104931_102202	Ga0104932_1002245<br>Ga0104932_100517	BZG02_01090<br>BZG02_03640	BZG01_02545<br>BZG01_04895
	2-oxoglutarate ferredoxin oxidoreductase subunit delta	KO:K00176	+	+	Ga0104931_102204	Ga0104932_100519	BZG02_03650	BZG01_04905
	2-oxoglutarate ferredoxin oxidoreductase subunit gamma	KO:K00177	+	+	Ga0104931_102201	Ga0104932_100516	BZG02_03635	BZG01_04890
	Citrate lyase subunit alpha / citrate CoA-transferase	TIGR01584	+	+	Ga0104931_106205<br>Ga0104931_11916	Ga0104932_102064<br>Ga0104932_102355	BZG02_10550<br>BZG02_17430	BZG01_13535<br>BZG01_14545
	Citrate lyase subunit beta / citryl-CoA lyase	TIGR01608	+	+	Ga0104931_106206	Ga0104932_102354	BZG02_10555	BZG01_14540
	Citrate lyase subunit gamma (acyl carrier protein)	TIGR01608	+	+	Ga0104931_11914	Ga0104932_102066	BZG02_17420	BZG01_13545
	Pyruvate:ferredoxin (flavodoxin) oxidoreductase	TIGR02176	+	+	Ga0104931_102267	Ga0104932_1005137	BZG02_03950	BZG01_05485
	Phosphoenolpyruvate synthase/pyruvate phosphate dikinase	COG0574	+	+	Ga0104931_102265	Ga0104932_1005135	BZG02_03940	BZG01_05475
	Phosphoenolpyruvate carboxylase	COG2352	+	+	Ga0104931_11630	Ga0104932_100456	BZG02_16470	BZG01_04035
**Pyruvate degradation/ formation of acetate, formate and hydrogen**								
	Pyruvate-formate lyase	COG1882	+	+	Ga0104931_10477<br>Ga0104931_1193<br>Ga0104931_12920	Ga0104932_100822<br>Ga0104932_10212	BZG02_07225<br>BZG02_17365<br>BZG02_19895	BZG01_07070<br>BZG01_13625
	Pyruvate:ferredoxin (flavodoxin) oxidoreductase	TIGR02176	+	+	Ga0104931_102267	Ga0104932_1005137	BZG02_03950	BZG01_05485
	Phosphotransacetylase	COG0280	+	+	Ga0104931_110130	Ga0104932_1004188	BZG02_14190	BZG01_04675
	Acetate kinase	COG0282	+	+	Ga0104931_110131	Ga0104932_1004187	BZG02_14195	BZG01_04670
	Acylphosphatase	COG1254	+	+	Ga0104931_10874	Ga0104932_101930	BZG02_12275	BZG01_12865
	AcetylCoA synthetase	COG0365	+	+	Ga0104931_104251	Ga0104932_100172	BZG02_08075	BZG01_00355

**Formation of propionate**							
Methylmalonyl-CoA mutase	KO:K01847	+	+	Ga0104931_108138<br>Ga0104931_108140	Ga0104932_101625<br>Ga0104932_101626	BZG02_12595<br>BZG02_12605	BZG01_11575<br>BZG01_11580
Methylmalonyl-CoA mutase	KO:K11942	+	+	Ga0104931_11345	Ga0104932_104720	BZG02_15385	BZG01_19640
Methylmalonyl-CoA epimerase	KO:K05606	+	+	Ga0104931_106211	Ga0104932_102345	BZG02_10580	BZG01_14505
Propionyl-CoA carboxylase alpha chain	KO:K01965	+	+	Ga0104931_10318	Ga0104932_100417	BZG02_04995	BZG01_03835
Propionyl-CoA carboxylase beta chain	KO:K01966	+	+	Ga0104931_10316<br>Ga0104931_106228	Ga0104932_100416<br>Ga0104932_102328	BZG02_04985<br>BZG02_10665	BZG01_03830<br>BZG01_14420
**Lactate degradation**							
Lactate dehydrogenase	COG1052	-	+	-	Ga0104932_1009105	-	BZG01_08175
Lactate dehydrogenase	KO:K18930	+	+	Ga0104931_10917<br>Ga0104931_10918	Ga0104932_10519	BZG02_12935<br>BZG02_12940	BZG01_20085
**Glycerol degradation**							
Glycerol kinase	COG0554	+	+	Ga0104931_10993<br>Ga0104931_10997	Ga0104932_103352<br>Ga0104932_10664	BZG02_13315<br>BZG02_13325	BZG01_17090<br>BZG01_21060
Glycerol-3-phosphate dehydrogenase	COG0578	+	+	Ga0104931_101467	Ga0104932_10277	BZG02_02305	BZG01_15340
**Monosaccharide degradation**							
Xylose isomerase	KO:K01805	+	+	Ga0104931_104139	Ga0104932_100879	BZG02_07525	BZG01_07360
Xylulokinase	KO:K00854	+	+	Ga0104931_104140	Ga0104932_100880	BZG02_07530	BZG01_07365
Ribulose-phosphate 3-epimerase	KO:K01783	+	+	Ga0104931_102271	Ga0104932_1005141	BZG02_03970	BZG01_05505
Phosphofructokinase	COG0205	+	+	Ga0104931_102153<br>Ga0104931_103193<br>Ga0104931_11652<br>Ga0104931_101434	Ga0104932_100331<br>Ga0104932_100989<br>Ga0104932_102120<br>Ga0104932_100227	BZG02_03420<br>BZG02_05865<br>BZG02_16580<br>BZG02_02145	BZG01_02835<br>BZG01_08095<br>BZG01_13720<br>BZG01_01465
L-arabinose isomerase	COG2160	-	+	-	Ga0104932_100922	-	BZG01_07780
L-rhamnose mutarotase	COG3254	-	+	-	Ga0104932_1007110	-	BZG01_06880
L-rhamnose isomerase	COG4806	-	+	-	Ga0104932_100780	-	BZG01_06735

N-Acetylglucosamine kinase	KOG1794	+	+	Ga0104931_102338<br>Ga0104931_105206	Ga0104932_10143<br>Ga0104932_103824	BZG02_04295<br>BZG02_09240	BZG01_10560<br>BZG01_18050
**Disaccharide degradation**							
Cellobiose phosphorylase	COG3459	+	+	Ga0104931_102112	Ga0104932_100945	BZG02_03230	BZG01_07895
Trehalose and maltose hydrolase	COG1554	+	+	Ga0104931_10338<br>Ga0104931_11747	Ga0104932_1001124<br>Ga0104932_102619	BZG02_05085<br>BZG02_16945	BZG01_00620<br>BZG01_15160
L-galactose dehydrogenase [EC:1.1.1.316]	KO:K17744	+	-	Ga0104931_103139	-	BZG02_05590	-
Beta-galactosidase GanA	COG1874	+	+	Ga0104931_10560	Ga0104932_100633<br>Ga0104932_101455	BZG02_08505	BZG01_05855<br>BZG01_10825
Beta-galactosidase, beta subunit	COG2731	+	+	Ga0104931_110155	Ga0104932_100496	BZG02_14310	BZG01_04235
Beta-galactosidase/beta-glucuronidase	COG3250	+	+	Ga0104931_10216<br>Ga0104931_102299<br>Ga0104931_103127<br>Ga0104931_103135<br>Ga0104931_11412<br>Ga0104931_11536<br>Ga0104931_11538<br>Ga0104931_11540<br>Ga0104931_11844	Ga0104932_100192<br>Ga0104932_100641<br>Ga0104932_100773<br>Ga0104932_1007106<br>Ga0104932_100847<br>Ga0104932_101843	BZG02_02760<br>BZG02_04105<br>BZG02_05530<br>BZG02_05570<br>BZG02_15680<br>BZG02_16190<br>BZG02_16200<br>BZG02_16210<br>BZG02_17230	BZG01_00465<br>BZG01_05900<br>BZG01_06700<br>BZG01_06860<br>BZG01_07195<br>BZG01_12640
Arabinogalactan endo-1,4-beta-galactosidase	COG3867	-	+	-	Ga0104932_10184<br>Ga0104932_10189	-	BZG01_12445<br>BZG01_12470
Galactokinase	COG0153	+	+	Ga0104931_104213<br>Ga0104931_107128	Ga0104932_102252	BZG02_07885<br>BZG02_11450	BZG01_14205
**Oxygen stress**							
Superoxide dismutase fe-mn family	COG0605	+	+	Ga0104931_101216<br>Ga0104931_101217	Ga0104932_1002251<br>Ga0104932_1002252	BZG02_01055<br>BZG02_01060	BZG01_02575<br>BZG01_02580
Catalase (peroxidase I)	COG0376	+	+	Ga0104931_104103	Ga0104932_100816	BZG02_07350	BZG01_07045
Superoxide reductase	COG2033	+	+	Ga0104931_11656	Ga0104932_102125	BZG02_16595	BZG01_13740

**Oxygen reduction**							
Cytochrome c oxidase cbb3-type subunit I/II	TIGR00780<br>TIGR00781	+	+	Ga0104931_101375	Ga0104932_100286	BZG02_01845	BZG01_01755
Cytochrome c oxidase, cbb3-type, subunit iii	TIGR00782	+	+	Ga0104931_101377	Ga0104932_100284	BZG02_01855	BZG01_01745
Cytochrome c oxidase subunit I (coxA)	KO:K02274	+	+	Ga0104931_101385	Ga0104932_100276	BZG02_01895	BZG01_01705
Cytochrome c oxidase subunit II (coxB)	KO:K02275	+	+	Ga0104931_101388	Ga0104932_100273	BZG02_01910	BZG01_01690
Cytochrome c oxidase subunit III (coxC)	KO:K02276	+	+	Ga0104931_101386	Ga0104932_100275	BZG02_01900	BZG01_01700
Cytochrome c oxidase subunit IV (coxD, ctaF)	KO:K02277	+	+	Ga0104931_101387	Ga0104932_100274	BZG02_01905	BZG01_01695
Cytochrome bd-type quinol oxidase, subunit 1	COG1271	-	+	-	Ga0104932_100294	-	BZG01_01790
Cytochrome bd-type quinol oxidase, subunit 2	COG1294	+	+	Ga0104931_101367	Ga0104932_100295	BZG02_01805	BZG01_01795
**Potential genes involved in metal reduction**							
Predicted ferric reductase	COG4097	+	-	Ga0104931_10793	-	BZG02_11275	-
Nitrite reductase (cytochrome c-552), NrfA	KO:K03385	+	+	Ga0104931_101168	Ga0104932_1005178	BZG02_00820	BZG01_05690
Cytochrome c nitrite reductase small subunit, NrfH	KO:K15876	+	+	Ga0104931_101169	Ga0104932_1005179	BZG02_00825	BZG01_05695

no_NCBI_match: indicates genes annotated by the IMG pipeline that were not identified by the NCBI Prokaryotic Genome Annotation Pipeline

1.2 Supplementary Figures

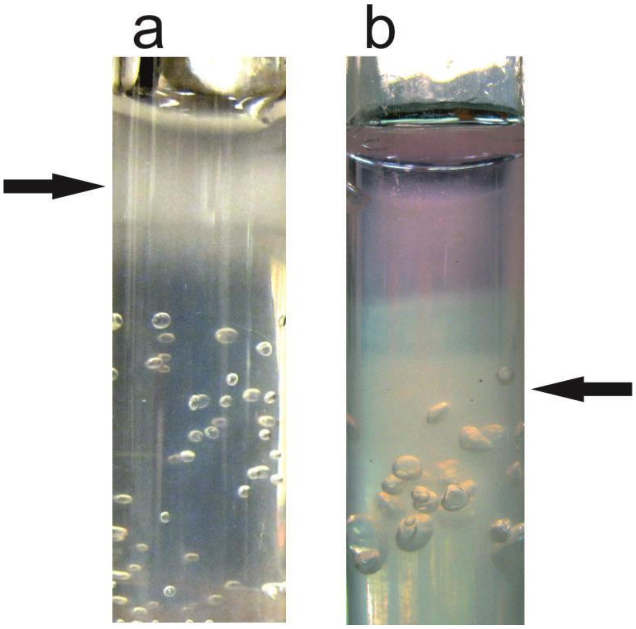

Supplementary Figure A.1. For tests on microaerophilic growth, cells were grown with 15 mM glucose in anoxic softagar and air in the headspace. (a) Cells of strain 59.10-2M localized preferably at the top of the tube little below the surface, which was in direct contact with air (whitish band indicated by arrow). (b) Tube with cells of strain 59.16B. Oxygen diffused into the top of the agar from the headspace indicated by pink coloration of resazurin, below a transparent zone where resazurin was already decolorized, but cells still did not grow, only below this zone cells grew (arrow) where anoxic conditions prevailed. Bubbles in the bottom of the tubes indicated hydrogen formation during fermentation of glucose.
